# Sex ratio at birth across 100 years in Sweden and risk of cardiovascular disease and all-cause mortality – a national register study

**DOI:** 10.1007/s10654-024-01137-1

**Published:** 2024-07-15

**Authors:** Peter M. Nilsson, Kristina Sundquist, Jan Sundquist, Casey Crump, Xinjun Li

**Affiliations:** 1 Department of Clinical Sciences Malmö, Lund University, Jan Waldenströms gata 15, floor 5, Malmö, Sweden; 2 Centre for Primary Health Care Research, Skane University Hospital, Malmö, S-20502 Sweden; 3 Departments of Family and Community Medicine and of Epidemiology, The University of Texas Health Science Centre, Houston, TX USA

**Keywords:** Cardiovascular disease, Epidemiology, Mortality, Registers, Sex ratio at birth, Time trends

## Abstract

The human sex ratio at birth (SRB) undergoes temporary changes around a mean proportion of 0.51 male births. SRB has been well studied for historical, geographical, and secular trends, but until now not linked to health outcomes in the total population, e.g. for cardiovascular disease (CVD) or mortality during follow-up of birth cohorts. We used linkage analysis based on national registers in Sweden that cover all births from 1900 to 2016. SRB at birth was calculated by every 10-year birth cohort in all survivors living in 1997 for a follow-up analysis of risk of CVD and mortality with data from national registers. When the highest quartile of SRB was used as reference, a slightly increased risk of fatal CVD (HR 1.03 (95% confidence intervals, CI): 1.02–1.04), non-fatal CVD (HR 1.01; 95%CI: 1.01–1.02) and mortality (HR 1.02; 95%CI, 1.01–1.03) was found after full adjustments in men belonging to the lowest SRB quartile. A similar pattern was also found for fatal CHD in women. in the lowest SBR quartile compared to the highest, HR 1.03 (95%CI: 1.02–1.05). In conclusion, in birth cohorts with a relatively lower than expected number of males born, long-term adverse health effects were observed with slightly increased cardiovascular risk and total mortality at the population level. This could indicate that men belonging to so-called “culled cohorts” in a developed country during the 20th century are characterized by a slightly increased risk that could reflect negative early life influences and environmental exposures in pregnant women resulting in selective loss of male embryos or fetuses. In a public health perspective SRB could be of some importance to monitor as an aspect of birth statistics linked to relatively minor population health effects.

**Supplementary Information:**

The online version contains supplementary material available at 10.1007/s10654-024-01137-1.

## Introduction

Cardiovascular disease (CVD) continues to be a major public health problem in western countries, even though age-adjusted trends for their incidence and mortality have been declining in many countries [1]. Despite corresponding age-adjusted declining trends for CVD risk factors, such as mean systolic blood pressure [2], total cholesterol [3], and smoking, the growth of ageing populations means that, in absolute terms, the number of CVD events will remain high.

Besides the well-known major conventional risk factors for CVD like older age, male sex, hypertension, hyperlipidaemia, obesity, diabetes and smoking, an increased interest has focused on the importance of *reproductive history* for evaluation of CVD risk, both in women [4–6] and in men [7]. For example, individual-level reproductive outcomes at birth such as prematurity, growth retardation, and low birth weight have been reported to predict CVD in adult life [8, 9]. Less well studied are population-level reproductive outcomes such as the so-called *secondary sex ratio at birth*, SRB (as opposed to the primary sex ratio at conception). SRB is a rather robust variable that is simple to measure over time [10]. Previous demographic studies have shown that, in general, the proportion of male births is about 0.51 of all births [11], which is slightly less than the proportion of male embryos that are initially conceived (primary sex ratio), due to unsuccessful implantations or spontaneous abortions [12].

The SRB and its secular trends over time have been a challenge in demographic research for many years [13–15] despite the relative simplicity of measuring them. This is because these trends may have been influenced by biological as well as social and cultural factors [16, 17]. As medical technologies evolve, pre-natal diagnosis based on ultrasound and selective abortions have been used to alter the natural SRB because in some countries having a male baby means securing the family name with implications for family economy [18, 19]. The SRB is currently declining in western countries [20, 21], including Sweden [22, 23], but increasing in other parts of the world [24]. However, underlying factors behind these secular trends and whether such trends are independently associated with a changing pattern of cardiovascular events and all-cause mortality are poorly understood.

Based on linkage of extensive national register data from Sweden our *primary aim* was to examine demographic change of SRB over a period of more than 100 years and its associations with risk of CVD overall, coronary heart disease (CHD), and total mortality, stratified by sex. *Secondary aims* included the description of SRB in relation to geographical regions and social as well as maternal background characteristics.

## Materials and methods

### Setting and participants

The dataset used in this study was constructed by linking several national Swedish registers. The Swedish government-owned Statistics Sweden provided the Nationwide Population Register and the Multi-Generation Register (MGR), which include persons born in Sweden in or after 1900 [25]. The MGR consists of data of more than nine million individuals, with information available on mothers in 97% and on fathers in 95% of index persons. Index persons are confined to those born from 1932 onwards and those alive on January 1, 1961 [25].

We calculated the SRB annually for all children born in Sweden from 1900 to 2016. Linkages were made to National Census data, available since 1960, to ascertain individual-level socioeconomic status. The final link was made by adding data from the Swedish Cause of Death Register (1961–2018) and the Swedish Hospital Discharge Register, with recorded dates of hospitalizations and hospital diagnoses since 1964, and almost complete national coverage since 1987, until the end of 2018. National Swedish registers have a high validity for medical research [26], including reproductive epidemiology [27, 28]. For analysing the risk of CVD, CHD, and total mortality in relation to SRB, we collected data from 4.07 million men and 4.14 million women who were born from 1900 to 1997 and still living in 1997, who were then followed up for the study outcomes through 2018.

### Patient and public involvement

No participants or patients were involved in setting the research question or in developing plans for the study. The results will be disseminated to patients and the public through a website and/or press releases suitable for a non-specialized audience.

### Follow-up of CVD/CHD events and total mortality

We used ICD-codes for non-fatal or fatal CVD (ICD-10, I00-I99) and CHD (ICD-10, I20-I25). Non-fatal events were identified from the National Hospital Discharge Register, and fatal events and total mortality from the Cause of Death Register, until 31st December 2018.

### Definitions

*Education* was based on educational level, which was classified into three categories: ≤ 9 years, 10–11 years, and ≥ 12 years.

*Geographic region* was divided into large cities (cities with a population of more than 200,000 inhabitants), other/Southern Sweden, and other/Northern Sweden.

*Co-morbidities* were defined as the first hospitalization during the follow-up period of the following diseases: *chronic obstructive pulmonary disease*, COPD (both hospitalization and mortality were included) (ICD-9 490–496 and ICD-10 J40-J49), *obesity* (ICD-9 278 A and ICD-10 E65-E68), *alcohol-related liver disease* (both hospitalizations and mortality were included) (ICD-9 291, 303, 571 and ICD-10 F10 and K70), *diabetes mellitus* (both hospitalizations and mortality were included) (ICD-9 250 and ICD-10 E10-E14), and cancer diagnoses (ICD-7 140–209).

*SRB* was defined as the proportion of all births that were male, categorized in three groups as the lowest quartile, middle 50%, and highest quartile, based on the distribution within each 10-year birth cohort (Supplementary Table).

### Statistical methods

Person-years at risk were calculated from the start of follow-up on 1st January 1997 until our outcomes, death from other causes, emigration, or the end of the follow-up on 31st December 2018. Age at baseline was included as a continuous variable in the adjustments. Thus, age-adjusted incidence rates for first hospitalization and mortality were calculated for the entire follow-up period. We used Cox proportional hazards models to calculate the hazard ratio (HR) with 95% confidence intervals (CI) for total (fatal and non-fatal) CVD and CHD event risk, and for total mortality, for both men and women, associated with the calculated SRB. Attained age was used as the underlying timescale to enable comparison between individuals of the same age at follow-up. This was done while adjusting for individual characteristics (family income, marital status, immigrant background and educational level, and region of residence), and co-morbidities (COPD, obesity, alcohol-related liver diseases, diabetes mellitus, and cancer). Individuals in the *highest SRB* category (i.e., highest quartile of the proportion of male to female births) were used as reference. The proportionality assumptions were checked by plotting the incidence rates over time and by calculating Schoenfeld (partial) residuals and these assumptions were fulfilled. We used SAS version 9.4 (SAS Institute Inc. Cary, NC, USA) for all statistical analyses.

In sensitivity analyses (based on a smaller study sample, *N* = 6 563 604, with available information), further adjustment was made for maternal age at delivery and total number of children (parity, family size), when the risk for the different outcomes was calculated. This was made because maternal age and parity are interlinked and that having more than 3 children in non-smoking women was associated with relatively less boys born than expected [29].

To assess the potential influence of survivor bias, two different time periods of births (1900 to 1934 and 1935 to 1980) were compared in a second sub-analysis. A *p*-value < 0.05 was considered statistically significant.

## Results

**Fig. 1:**
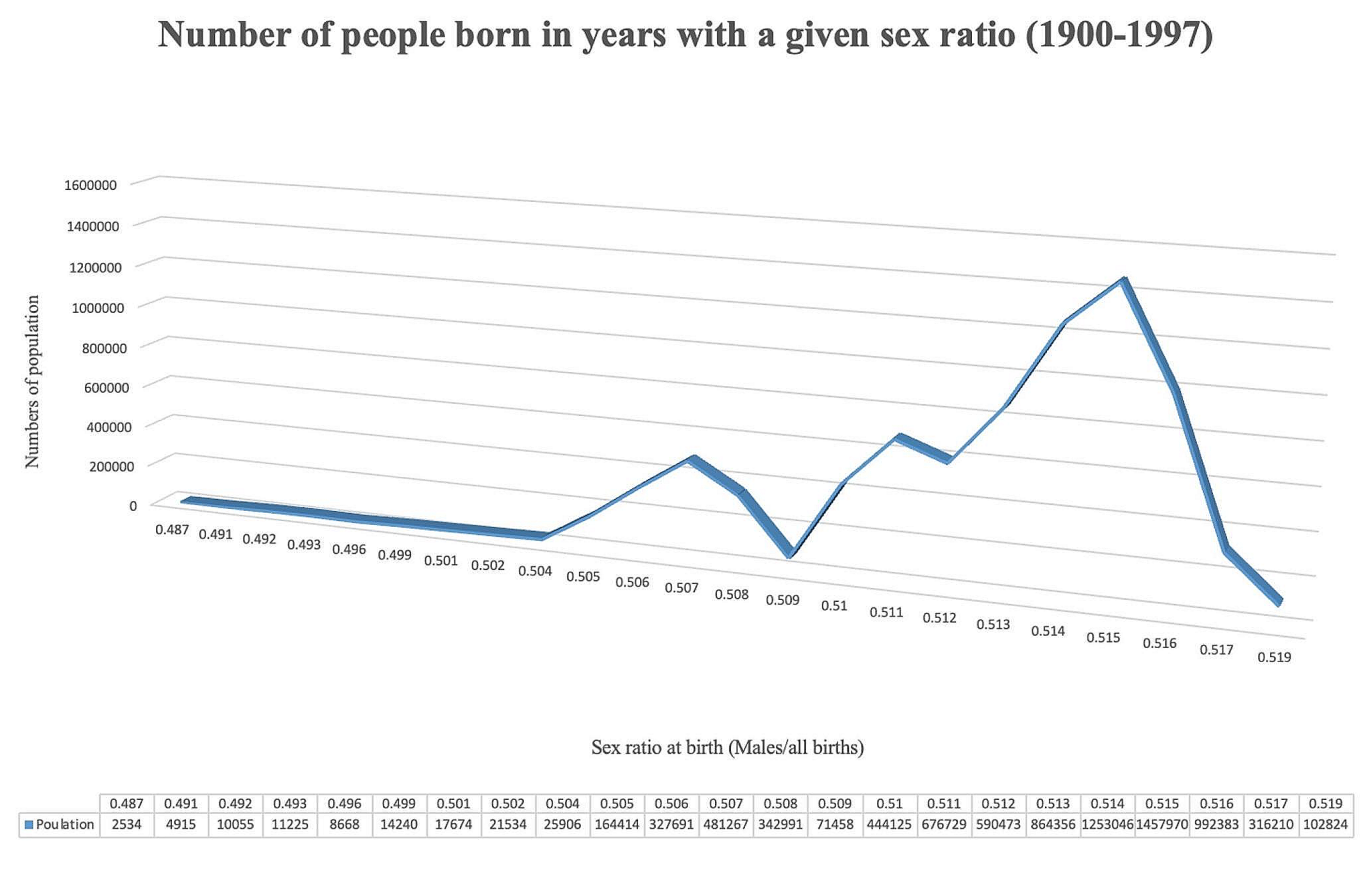
Study population by sex ratio (males/all birth) at birth 1900–1997 in Sweden

There was a total of 10,081,447 registered births (51.1% men) between 1900 and 1997 (Fig. 1). Among 8,202,688 people still living in 1997, 49.6% were men. From 1997 to 2018, there was a total of 2,716,574 incident CVD events, of which 873,149 were fatal events. Correspondingly there were 463,369 incident CHD events and 340,796 deaths from CHD. Total mortality included 1,802,452 deaths (Table 1).

**Table 1:** Study population (birth year 1900–1997) and number of cases of CHD. CVD. And fatal CHD. Fatal CVD and total mortality. 1997–2018

	Total population			Number of events of CHD			Number of events of CVD			Number of fatal CHD			Number of fatal CVD			Number of total mortality	
	No.	%		No.	%		No.	%		No.	%		No.	%		No.	%
Sex ratio at birth (Males/All birth)																	
Low	2,306,840	28.1		123,889	26.7		727,380	26.8		95,747	28.1		245,759	28.1		503,384	27.9
Middle	4,081,265	49.8		228,954	49.4		1,337,876	49.2		171,157	50.2		439,406	50.3		899,137	49.9
High	1,814,583	22.1		110,526	23.9		651,318	24.0		73,892	21.7		187,984	21.5		399,931	22.2
Age (years)																	
< 45	4,737,883	57.8		46,535	10.0		654,191	24.1		8711	2.6		22,813	2.6		99,357	5.5
45–54	1,154,566	14.1		84,788	18.3		532,786	19.6		23,168	6.8		51,682	5.9		157,233	8.7
55–64	805,667	9.8		97,920	21.1		510,699	18.8		42,243	12.4		101,458	11.6		260,599	14.5
65–74	708,284	8.6		113,726	24.5		516,619	19.0		96,506	28.3		250,627	28.7		513,890	28.5
≥ 75	796,288	9.7		120,400	26.0		502,279	18.5		170,168	49.9		446,569	51.1		771,373	42.8
Sex																	
Men	4,065,146	49.6		282,384	60.9		1,356,880	49.9		186,753	54.8		420,933	48.2		877,867	48.7
Women	4,137,542	50.4		180,985	39.1		1,359,694	50.1		154,043	45.2		452,216	51.8		924,585	51.3
Total	8,202,688	100.0		463,369	100.0		2,716,574	100.0		340,796	100.0		873,149	100.0		1,802,452	100.0

CHD: Coronary heart disease: CVD: Cardiovascular diseases

Almost half of the subjects lived in large cities (49.7%) and had a lower education level, 9 years or less (49.8%) (Supplementary Tables S1 and S2).

**Fig. 2:**
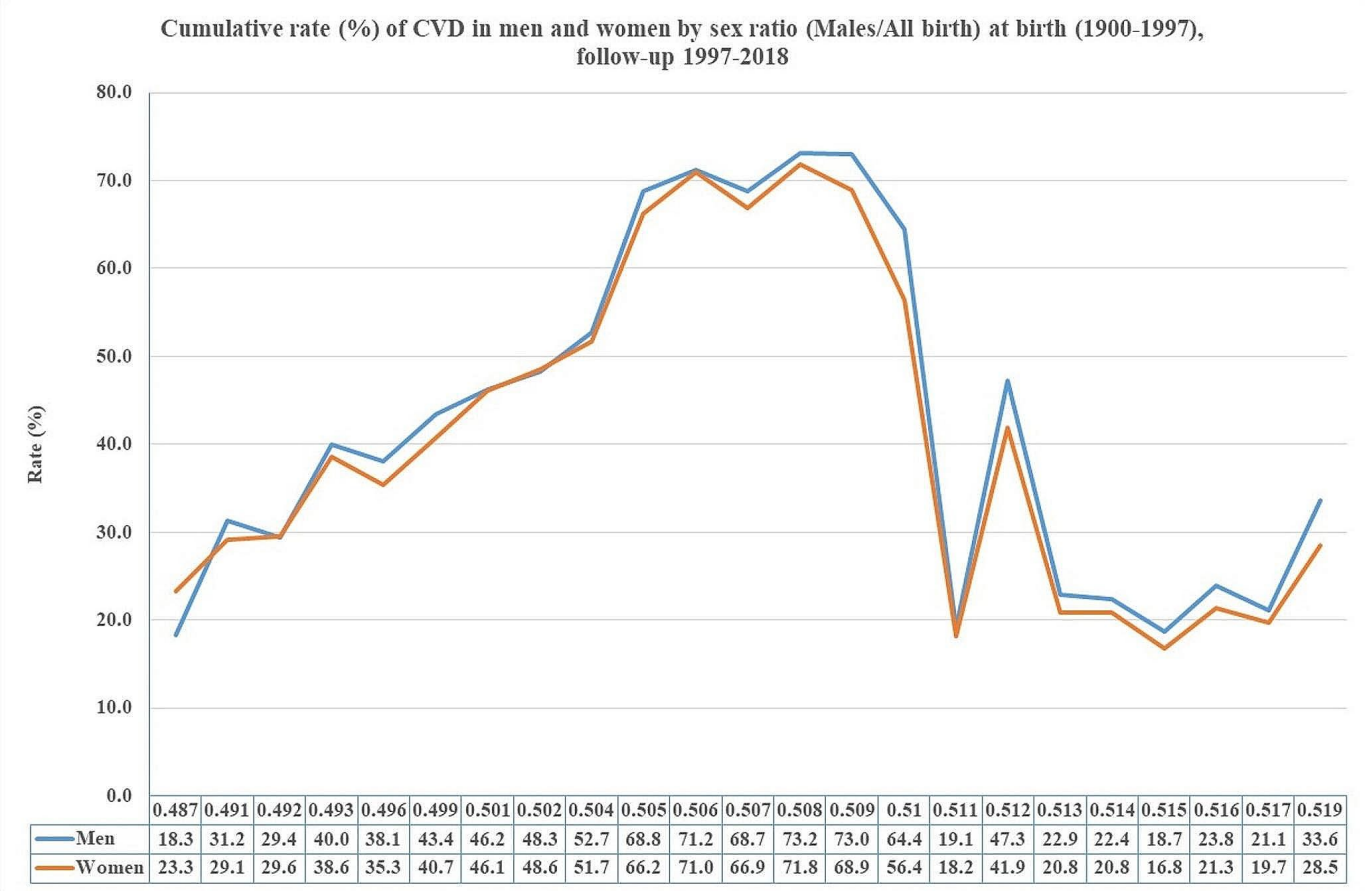
Cumulative rates of non-fatal CVD in men and women by sex ratio (males/all birth) at birth 1900–1997). follow-up 1997–2018 in Sweden

A shifting trend appeared for the cumulative rates of non-fatal CVD in men and women by sex ratio (males/all births) at birth 1900–1997 and during the follow-up 1997–2018 (Fig. 2).

### Risk of CVD and CHD in men in relation to sex ratio at birth

The risk of *non-fatal CVD* in men born in those years with the lowest SRB compared to the highest (reference) was slightly but significantly higher, HR 1.01 (95% CI: 1.01–1.02) when adjusted for age, region of residence and educational level, and remained significant HR 1.01 (1.01–1.02) when also adjusted for comorbidities, i.e. hospitalization for COPD, alcoholism, diabetes, obesity, and cancer (Table 2, supplementary Table S5). A similar risk was also noted for men in the middle SBR category after full adjustment, HR 1.01 (1.00-1.01).

A similar pattern was observed for *non-fatal CHD* in men in the lowest category of SRB with a HR of 1.03 (1.02–1.05) when adjusted for age, region of residence and educational level, and remained significant with a HR of 1.03 (1.02–1.04) after full adjustment for co-morbidities (Table 2, supplementary Table S6). For men in the middle SBR category, the HR was 1.02 (1.01–1.03) after full adjustment.

The risk of *fatal CVD* in men born in years with the lowest SRB compared to the highest was weakly but significantly higher with a HR of 1.03 (95% CI: 1.02–1.04) when adjusted for age, region of residence, and educational level, and remained significant, HR 1.03 (1.02–1.04), when also adjusted for comorbidities (Table 3, supplementary Table S3). A similar risk was noted for men in the middle SBR category after full adjustment with a HR of 1.03 (1.02–1.03).

**Table 2:** Association between sex ratio (males/all birth) at birth (1900–1997) and risks of non-fatal CVD and CHD, 1997–2018

	Model 1				Model 2				Model 3			
	HR	95% CI			HR	95% CI			HR	95% CI		
**Males CVD**												
Sex ratio at birth (Males/All birth) (ref. High)												
Low	1.01	1.01	1.02		1.01	1.01	1.02		1.01	1.01	1.02	
Middle	1.00	1.00	1.01		1.00	1.00	1.01		1.01	1.00	1.01	
**Males CHD**												
Sex ratio at birth (Males/All birth) (ref. High)												
Low	1.04	1.03	1.05		1.03	1.02	1.05		1.03	1.02	1.04	
Middle	1.02	1.01	1.03		1.02	1.01	1.03		1.02	1.01	1.03	
**Females CVD**												
Sex ratio at birth (Males/All birth) (ref. High)												
Low	1.00	1.00	1.01		1.00	1.00	1.01		1.00	1.00	1.00	
Middle	0.99	0.99	1.00		1.00	0.99	1.00		1.00	0.99	1.00	
**Females CHD**												
Sex ratio at birth (Males/All birth) (ref. High)												
Low	1.03	1.02	1.05		1.03	1.01	1.04		1.02	1.01	1.04	
Middle	1.02	1.01	1.04		1.03	1.01	1.04		1.03	1.01	1.04	

CVD: Cardiovascular diseases; CHD: Coronary heart disease; HR: Hazards ratio; CI: Confidence interval.

Model 1. Crude model; Model 2: Adjusted for individual characteristics (family income, marital status, immigrant background and educational level, and region of residence); Model 3: Fully adjusted model: Adjusted for individual characteristics and co-morbidities (COPD, obesity, alcohol-related liver diseases, diabetes mellitus, and cancer)

**Table 3:** Association between sex ratio (Males/all birth) at birth (1900–1997) and risks of fatal CVD. CHD and total mortality 1997–2018

	Model 1				Model 2				Model 3		
	HR	95% CI			HR	95% CI			HR	95% CI	
**Males fatal CVD**											
Sex ratio at birth (Males/All birth) (ref. High)											
Low	1.03	1.03	1.04		1.03	1.02	1.04		1.03	1.02	1.04
Middle	1.03	1.02	1.04		1.03	1.02	1.04		1.03	1.02	1.03
**Males fatal CHD**											
Sex ratio at birth (Males/All birth) (ref. High)											
Low	1.05	1.04	1.06		1.04	1.03	1.05		1.04	1.03	1.05
Middle	1.03	1.02	1.05		1.04	1.02	1.05		1.03	1.02	1.05
**Males total mortality**											
Sex ratio at birth (Males/All birth) (ref. High)											
Low	1.03	1.02	1.03		1.02	1.01	1.03		1.02	1.01	1.03
Middle	1.02	1.01	1.02		1.02	1.01	1.02		1.02	1.01	1.02
**Females fatal CVD**											
Sex ratio at birth (Males/All birth) (ref. High)											
Low	1.02	1.01	1.02		1.01	1.00	1.02		1.01	1.00	1.02
Middle	1.02	1.01	1.03		1.02	1.01	1.03		1.02	1.01	1.03
**Females fatal CHD**											
Sex ratio at birth (Males/All birth) (ref. High)											
Low	1.04	1.02	1.05		1.03	1.02	1.05		1.03	1.02	1.05
Middle	1.04	1.03	1.05		1.04	1.03	1.06		1.04	1.03	1.06
**Females total mortality**											
Sex ratio at birth (Males/All birth) (ref. High)											
Low	1.01	1.00	1.02		1.01	1.00	1.01		1.00	1.00	1.01
Middle	1.01	1.00	1.01		1.01	1.00	1.01		1.01	1.01	1.02

CVD: Cardiovascular diseases; CHD: Coronary heart disease; HR: Hazards ratio; CI: Confidence interval.

Model 1. Crude model; Model 2: Adjusted for individual characteristics (family income, marital status, immigrant background and educational level, and region of residence); Model 3: Fully adjusted model: Adjusted for individual characteristics and co-morbidities (COPD, obesity, alcohol-related liver diseases, diabetes mellitus, and cancer)

The risks of *fatal CHD* in men born in years with the lowest or middle SBR categories after full adjustment were also modestly higher with HRs of 1.04 (1.03–1.05) and 1.03 (1.02–1.05), respectively (Table 3, supplementary Table S4).

### Risk of CVD and CHD in women in relation to sex ratio at birth

The corresponding risk of *non-fatal CVD* in women with the lowest SRB compared to the highest (reference) was not increased with a HR of 1.00 (95% CI: 1.00–1.01) when adjusted for age, region of residence and educational level, and remained with a HR of 1.00 (1.00–1.00) when also adjusted for comorbidities (Table 2, supplementary Table S9). A similar risk was also noted for women in the middle SBR category after full adjustment, HR 1.00 (0.99-1.00).

For *non-fatal CHD* in women in the lowest SBR categories, the HRs were 1.03 (1.02–1.05) and 1.02 (1.01–1.04), respectively (Table 2, supplementary Table S10). The risk in women in the middle SBR category was similar after full adjustment with a HR of 1.03 (1.01–1.04).

The risk of *fatal CVD* in women born in years with the lowest SRB category compared to the highest was weakly but significantly higher with a HR of 1.01 (95% CI: 1.00-1.02) when adjusted for age, region of residence and educational level, and remained significant, HR 1.01 (1.00-1.02) when also adjusted for comorbidities (Table 3, supplementary Table S7). For women in the middle SBR category the risk was similar after full adjustment with a HR of 1.02 (1.01–1.03).

Weakly increased risks were also observed for *fatal CHD* in women in the lowest SBR category with HRs of 1.03 (1.02–1.05) and 1.03 (1.02–1.05) after full adjustment, respectively (Table 3, supplementary Table S8). For women in the middle SBR category the risk was similar after full adjustment with HR 1.04 (1.03–1.06).

### Risk of all-cause mortality in men and women in relation to sex ratio at birth

Men born in years with the lowest SRB category had slightly increased total mortality compared to the highest SRB category (HR, 1.02; 95% CI, 1.01–1.03) after full adjustment, as had men born in the middle SRB category, HR 1.02 (1.01–1.02). Women born in years with the lowest or middle SRB category had almost no increased total mortality risk compared to the highest SRB category (HR, 1.00; 95% CI, 1.00-1.01; and 1.01; 1.01–1.02, respectively) (Table 3, supplementary Tables S11 and S12).

### Sensitivity analyses after adjustment for maternal age at birth and family size

In a subsample based on 6,563,604 births, a further adjustment was made for family size (number of children) and maternal age at birth. The risk of *non-fatal CVD* in both men and women in the lowest SRB category compared to the highest was unchanged (HR, 1.01; 95% CI: 1.00-1.01). The corresponding risk for *non-fatal* CHD was, however, significantly lower in men (HR, 0.95; 95% CI, 0.94–0.97) and in women (0.93; 0.91–0.95) (supplementary Table S13).

For *fatal CVD* in men born in years with the lowest SRB compared to the highest the HR was significantly lower at 0.93 (95% CI: 0.92–0.95) and for women the HR was 0.96 (0.94–0.99). The corresponding risk for fatal CHD was lower in both men with a HR of 0.92 (0.90–0.94) and in women with a HR of 0.91 (0.87–0.95).

Finally, the corresponding risk for *total mortality* in men in the lowest SBR category was significantly lower with a HR of 0.98 (0.97–0.99), but non-significant in women with a HR of 1.01 (0.99–1.02) (supplementary Table S14).

### Sensitivity analyses in relation to period and gender

In a further sensitivity analysis (Table S15), it was shown that cardiovascular outcomes were similar between two separate time periods comparing children born 1900–1934 with children born 1935–1980, except for total mortality, with a higher risk observed in men during the first period RR 1.12 (95% CI: 1.11–1.13) compared to the second period, RR 1.04 (1.02–1.05), (Table S16). The risk ratio for men versus women was higher for all fatal outcomes (Table S17).

## Discussion

The most important finding of this register-based study that spanned over a period of almost 100 years in Sweden was the association between a lower SRB (i.e. fewer boys born than expected) and slightly increased risks of cardiovascular and coronary events (non-fatal and fatal) in both men and women during the follow-up and after adjustment for several background factors including comorbidities. However, for total mortality, the risk was higher in men but almost unchanged in women belonging to the same lowest SBR category. These findings are observational and admittedly quite weak. Their underlying explanation cannot be conclusively determined; thus, they are primarily hypothesis-generating. In sensitivity analyses, some of these findings became reduced or even reversed (i.e. showing lower, not higher risk associated with lower SRB) after adjustment for maternal age at delivery and parity status. This could imply that these maternal factors are associated with SRB in a way that increases the risk associated with low SRB. For example, during periods of adverse environmental influences, such as financial recession, unemployment, or shortage of material resources, women may both postpone childbearing until later during their reproductive life (older age), and have fewer children, thereby affecting parity status [30, 31].

Furthermore, two time periods were compared as well as the relative risk for men and women. The findings indicated a similar risk for cardiovascular events during the two periods, but a higher mortality risk in men from the lowest sex ratio at birth category during the first period (1900–1934) than during the second period (1935–1980). In general, the relative risk for fatal events in men was higher than that for women during both periods.

During this long period Sweden underwent a transition from a country dominated by agriculture and a larger part of the population living in rural areas to a highly developed industrialised country with most people living in urban areas. Important social changes took place with women entering the labour market in the 1960s and family size gradually decreasing with a lower number of children in each family. Other time trends include the demographic changes with increasing lifespan and more elderly people surviving into age ranges when cardiovascular and coronary disease become more prevalent.

No previous study, to the best of our knowledge, has addressed the same research question on trends for associations between SRB and cardiovascular outcomes as well as total mortality in both sexes following adjustment for several background factors. However, the association between SRB oscillations and longevity in men has previously been studied in some historical studies from the Nordic area [32–34]. These studies have indicated that adverse exogenous influences during gestation such as temperature changes or stress exposure could cause an increased abortion rate of the weaker male foetuses (“culled birth cohorts”) resulting in a lower SRB [32–34]. On the other hand, the male babies belonging to these “culled birth cohorts” during mostly pre-industrial historical periods (before 1913) who had less cardiovascular morbidity and mortality were selected for survival and, in addition, also had a longer life expectancy in unadjusted analyses, probably due to selection bias [33]. Catalano *et al*. suggested that oscillation of SRB appears to be due to social processes rather than heritable mechanisms [34]. This agrees with a modern study from Sweden reporting that environmental factors are more important determinants of SRB than genetic factors [17]. Another indication of environmental influences of SRB are studies in immigrant populations showing variation of SRB in relation to parity, country of origin, and time trends [35]. Finally, parental occupation has also been linked to various patterns of SRB [36, 37]. Taken together, these studies emphasise the importance of environmental factors for trends in SRB (Trivers-Willard Hypothesis) [38] acting through mechanisms during gestation including lack of proper implantation of male embryos or spontaneous abortions of male foetuses during adverse conditions related to external exposures during pregnancy. This might be of importance for cardiovascular risk in the offspring as maternal risk factors such as pre-pregnancy hypertension could influence offspring risk of hypertension, which also has been associated with a variation in SRB [39].

Strengths of the study include complete nationwide coverage from 1997 for outcomes in a country with high standards of diagnosis, and with diagnoses often being made by clinical specialists. Another important strength of our study is that it was based on nationwide registers and thus free of recall bias. The Swedish MGR and the Swedish Hospital Discharge Register are validated data sources that have been proven to be reliable in studies of many diseases [25, 26]. Data in our dataset are almost 100% complete [25] and were used for a previous study of ours evaluating sibling rank in relation to cardiovascular risk and total mortality [28].

A potential limitation is the possibility of survivor bias in people who lived until the beginning of follow-up in 1997. However, a sensitivity analysis yielded similar risk estimates in people born before or after 1935, suggesting that survivor bias did not strongly influence the main findings. Another limitation is that we had no access to data on individual lifestyle-related factors such as body mass index, smoking, or diet, because it is impossible to gather such data for an entire national population. However, we did adjust for socioeconomic status, obesity, diabetes, COPD, and cancer as well as alcoholism and related liver disorders, which are associated with lifestyle factors. No data reflecting psychology or personality traits were available to us.

Future research should be directed to find and characterize biological or environmental/social mechanisms linking low SRB to a higher individual risk of CVD, CHD, and mortality [40], as suggested by our observational findings. SRB has been shown to be lowered by several environmental factors and stressors, such as terrorist attacks during pregnancy [41], different catastrophes [42], and the Covid-19 pandemic [43]. This lends further evidence for the importance of the developmental origin of adult health (DoHAD) hypothesis with its many applications in preventive cardiology [44]. These associations could be further studied in other high-quality registers that can be linked by personal ID number in the Nordic countries where secular time trends of SRB during the 20th century have been well described [45].

## Conclusion

Based on national registers covering more than a century in Sweden we have shown that a lower SRB, i.e., fewer males than expected being born during some periods (“culled cohorts”), is associated with a slightly increased cardiovascular and coronary risk during follow-up, especially for men. For total mortality, the risk was higher in men, but unchanged in women, for those born during years with a lower SRB. This points to the influence of still undefined environmental factors of importance during gestation that regulate male survival, i.e. lack of implantation of male embryos or spontaneous abortion of male foetuses, with its further implications for reduced longevity in men and increased risk of cardiovascular disease. In addition, maternal characteristics such as age at pregnancy and parity seem to be of importance but could also be influenced by adverse environmental factors during periods of financial recession and lack of resources. Although the observed effects were very weak in general, they could still be of interest at the population level. In a public health perspective, SRB could be of some importance to monitor as an aspect of birth statistics linked to relatively minor population health effects.

## Electronic supplementary material

Below is the link to the electronic supplementary material.

Supplementary Material 1

## Data Availability

No additional data are available. The manuscript guarantors affirm that this manuscript is an honest, accurate, and transparent account of the study being reported; that no important aspects of the study have been omitted.

## Abbreviations

CHD: Coronary Heart Disease
COPD: Chronic Obstructive Pulmonary Disease
CVD: Cardiovascular disease
DOHaD: Developmental Origins of Health and Disease
SRB: Sex Ratio at Birth
