# Supplementary material for: Sex ratio at birth across 100 years in Sweden and risk of cardiovascular disease and all-cause mortality – a national register study
Source: Eur J Epidemiol. 2024 Jul 15;39(9):967–76. doi: 10.1007/s10654-024-01137-1 (PMC11470864; doi:10.1007/s10654-024-01137-1)
Supplement: Supplementary file 1 — Supplementary Material 1 [file 10654_2024_1137_MOESM1_ESM.docx]

**Supplementary materials:**

**Figure S1.** Sex ratio (males/all births) by birth years in Sweden 1900-1997.

**Table S1.** Study population (birth year 1900-1997) and number of cases of non-fatal CVD and CHD 1997-2018.

**Table S2.** Study population (birth year 1900-1997) and number of cases of fatal CVD, fatal CHD and total mortality 1997-2018.

**Table S3**. Association between sex ratio (males/all births) at birth (1900-1997) and risks of fatal CVD in men 1997-2018.

**Table S4.** Association between sex ratio (males/all births) at birth (1900-1997) and risks of fatal CHD in men 1997-2018.

**Table S5.** Association between sex ratio (males/all births) at birth (1900-1997) and risks of non-fatal CVD in men 1997-2018.

**Table S6.** Association between sex ratio (males/all births) at birth (1900-1997) and risks of non-fatal CHD in men 1997-2018.

**Table S7.** Association between sex ratio (males/all births) at birth (1900-1997) and risks of fatal CVD in women 1997-2018.

**Table S8.** Association between sex ratio (males/all births) at birth (1900-1997) and risks of fatal CHD in women 1997-2018.

**Table S9.** Association between sex ratio (males/all births) at birth (1900-1997) and risks of non-fatal CVD in women 1997-2018.

**Table S10.** Association between sex ratio (males/all births) at birth (1900-1997) and risks of non-fatal CHD in women 1997-2018.

**Table S11.** Association between sex ratio (males/all births) at birth (1900-1997) and total mortality in men 1997-2018.

**Table S12.** Association between sex ratio (males/all births) at birth (1900-1997) and risks of total mortality in women 1997-2018.

**Table S13.** Association between sex ratio (males/all births) at birth (1932-1997) and risks of non-fatal CVD and CHD 1997-2018, adjusted for family size and maternal age at birth (N=6 563 604)

**Table S14.** Association between sex ratio (males/all births) at birth (1932-1997) and risks of fatal CVD, fatal CHD, and total mortality 1997-2018, adjusted for family size and maternal age at birth (N=6 563 604)

**Table S15**. Numbers of births and outcomes during two separate time periods of birth and follow-up.

**Table S16.** Association between tertiles of sex ratio (males/all birth) at birth and risks of cardiovascular disorders and mortality during two time periods (1900-1934 vs. 1935-1980).

**Table S17.** Associations between tertiles of sex ratio (males/all birth) at birth (1900-1997) and risks of disease by gender, and risk ratio for men compared to women.

**Figure S1**


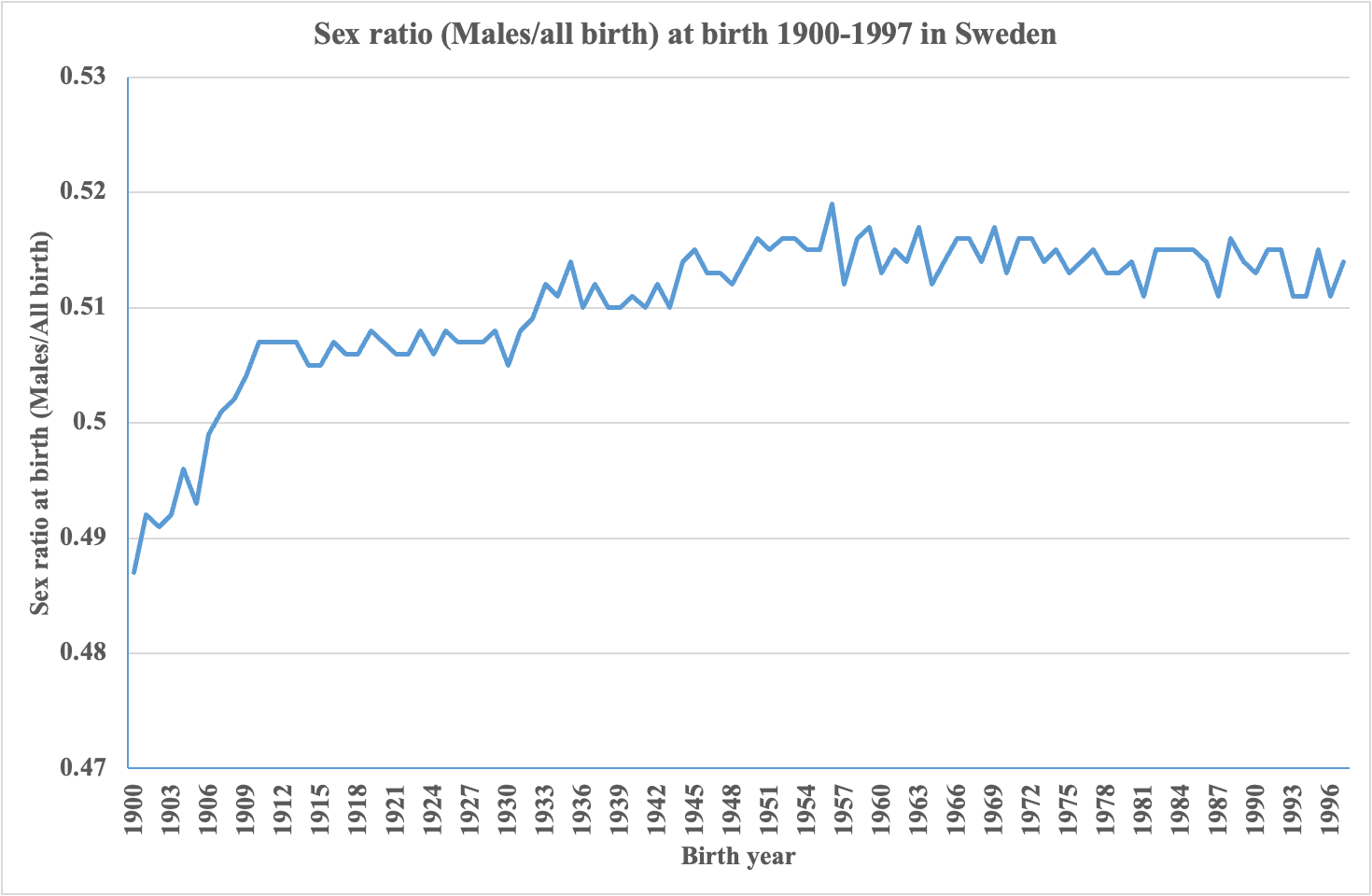


| **Table S1.** Study population (birth year 1900-1997) and number of cases of CHD and CVD during 1997-2018 | | | | | | | |  |
| --- | --- | --- | --- | --- | --- | --- | --- | --- |
|  | Total population | |  | Number of events of CHD |  |  | Number of events of CVD | |
|  | No. | % |  | No. | % |  | No. | % |
| Sex ratio at birth (Males/All birth) |  |  |  |  |  |  |  |  |
| Low | 2306840 | 28.1 |  | 123889 | 26.7 |  | 727380 | 26.8 |
| Middle | 4081265 | 49.8 |  | 228954 | 49.4 |  | 1337876 | 49.2 |
| High | 1814583 | 22.1 |  | 110526 | 23.9 |  | 651318 | 24.0 |
| Age (years) |  |  |  |  |  |  |  |  |
| <45 | 4737883 | 57.8 |  | 46535 | 10.0 |  | 654191 | 24.1 |
| 45-54 | 1154566 | 14.1 |  | 84788 | 18.3 |  | 532786 | 19.6 |
| 55-64 | 805667 | 9.8 |  | 97920 | 21.1 |  | 510699 | 18.8 |
| 65-74 | 708284 | 8.6 |  | 113726 | 24.5 |  | 516619 | 19.0 |
| ≥75 | 796288 | 9.7 |  | 120400 | 26.0 |  | 502279 | 18.5 |
| Sex |  |  |  |  |  |  |  |  |
| Men | 4065146 | 49.6 |  | 282384 | 60.9 |  | 1356880 | 49.9 |
| Women | 4137542 | 50.4 |  | 180985 | 39.1 |  | 1359694 | 50.1 |
| Region of residence |  |  |  |  |  |  |  |  |
| Large cities | 4073279 | 49.7 |  | 203474 | 43.9 |  | 1294712 | 47.7 |
| Southern Sweden | 2741197 | 33.4 |  | 164192 | 35.4 |  | 939061 | 34.6 |
| Northern Sweden | 1388212 | 16.9 |  | 95703 | 20.7 |  | 482801 | 17.8 |
| Education level |  |  |  |  |  |  |  |  |
| <9 | 4086958 | 49.8 |  | 229117 | 49.4 |  | 1205479 | 44.4 |
| 10-12 | 1754855 | 21.4 |  | 113187 | 24.4 |  | 707727 | 26.1 |
| >12 | 2360875 | 28.8 |  | 121065 | 26.1 |  | 803368 | 29.6 |
| Hospital diagnosis of COPD |  |  |  |  |  |  |  |  |
| Non | 7771720 | 94.7 |  | 425132 | 91.7 |  | 2511810 | 92.5 |
| Yes | 430968 | 5.3 |  | 38237 | 8.3 |  | 204764 | 7.5 |
| Hospital diagnosis of alcoholism |  |  |  |  |  |  |  |  |
| Non | 7946199 | 96.9 |  | 451308 | 97.4 |  | 2622480 | 96.5 |
| Yes | 256489 | 3.1 |  | 12061 | 2.6 |  | 94094 | 3.5 |
| Hospital diagnosis of diabetes |  |  |  |  |  |  |  |  |
| Non | 7869392 | 95.9 |  | 411692 | 88.8 |  | 2479915 | 91.3 |
| Yes | 333296 | 4.1 |  | 51677 | 11.2 |  | 236659 | 8.7 |
| Hospital diagnosis of obesity |  |  |  |  |  |  |  |  |
| Non | 8084044 | 98.6 |  | 459266 | 99.1 |  | 2670029 | 98.3 |
| Yes | 118644 | 1.4 |  | 4103 | 0.9 |  | 46545 | 1.7 |
| Diagnosis of cancer |  |  |  |  |  |  |  |  |
| Non | 7275765 | 88.7 |  | 368520 | 79.5 |  | 2168005 | 79.8 |
| Yes | 926923 | 11.3 |  | 94849 | 20.5 |  | 548569 | 20.2 |
| All | 8202688 | 100.0 |  | 463369 | 100.0 |  | 2716574 | 100.0 |
| CHD: Coronary heart disease: CVD: Cardiovascular diseases; COPD: Chronic obstructive pulmonary disease. | | | | | | | | |

| **Table S2**. Study population (birth year 1900-1997) and number of fatal CVD. CHD and total mortality during 1997-2018 | | | | | | | | | | | |
| --- | --- | --- | --- | --- | --- | --- | --- | --- | --- | --- | --- |
|  | Total population | |  | Number of fatal CHD | |  | Number of fatal CVD | |  | Number of total mortality | |
|  | No. | % |  | No. | % |  | No. | % |  | No. | % |
| Sex ratio at birth (Males/All birth) |  |  |  |  |  |  |  |  |  |  |  |
| Low | 2145250 | 26.1 |  | 95747 | 28.1 |  | 245759 | 28.1 |  | 503384 | 27.9 |
| Middle | 4032708 | 49.2 |  | 171157 | 50.2 |  | 439406 | 50.3 |  | 899137 | 49.9 |
| High | 2024730 | 24.7 |  | 73892 | 21.7 |  | 187984 | 21.5 |  | 399931 | 22.2 |
| Age (years) |  |  |  |  |  |  |  |  |  |  |  |
| <45 | 4737883 | 57.8 |  | 8711 | 2.6 |  | 22813 | 2.6 |  | 99357 | 5.5 |
| 45-54 | 1154566 | 14.1 |  | 23168 | 6.8 |  | 51682 | 5.9 |  | 157233 | 8.7 |
| 55-64 | 805667 | 9.8 |  | 42243 | 12.4 |  | 101458 | 11.6 |  | 260599 | 14.5 |
| 65-74 | 708284 | 8.6 |  | 96506 | 28.3 |  | 250627 | 28.7 |  | 513890 | 28.5 |
| ≥75 | 796288 | 9.7 |  | 170168 | 49.9 |  | 446569 | 51.1 |  | 771373 | 42.8 |
| Sex |  |  |  |  |  |  |  |  |  |  |  |
| Men | 4065146 | 49.6 |  | 186753 | 54.8 |  | 420933 | 48.2 |  | 877867 | 48.7 |
| Women | 4137542 | 50.4 |  | 154043 | 45.2 |  | 452216 | 51.8 |  | 924585 | 51.3 |
| Region of residence |  |  |  |  |  |  |  |  |  |  |  |
| Large cities | 4073279 | 49.7 |  | 160807 | 47.2 |  | 414895 | 47.5 |  | 870979 | 48.3 |
| Southern Sweden | 2741197 | 33.4 |  | 116207 | 34.1 |  | 299861 | 34.3 |  | 608517 | 33.8 |
| Northern Sweden | 1388212 | 16.9 |  | 63782 | 18.7 |  | 158393 | 18.1 |  | 322956 | 17.9 |
| Education level |  |  |  |  |  |  |  |  |  |  |  |
| <9 | 4086958 | 49.8 |  | 204621 | 60.0 |  | 513611 | 58.8 |  | 1015477 | 56.3 |
| 10-12 | 1754855 | 21.4 |  | 60875 | 17.9 |  | 158013 | 18.1 |  | 356734 | 19.8 |
| >12 | 2360875 | 28.8 |  | 75300 | 22.1 |  | 201525 | 23.1 |  | 430241 | 23.9 |
| Hospital diagnosis of COPD |  |  |  |  |  |  |  |  |  |  |  |
| Non | 7771720 | 94.7 |  | 315972 | 92.7 |  | 809993 | 92.8 |  | 1658422 | 92.0 |
| Yes | 430968 | 5.3 |  | 24824 | 7.3 |  | 63156 | 7.2 |  | 144030 | 8.0 |
| Hospital diagnosis of alcoholism |  |  |  |  |  |  |  |  |  |  |  |
| Non | 7946199 | 96.9 |  | 330525 | 97.0 |  | 849322 | 97.3 |  | 1732464 | 96.1 |
| Yes | 256489 | 3.1 |  | 10271 | 3.0 |  | 23827 | 2.7 |  | 69988 | 3.9 |
| Hospital diagnosis of diabetes |  |  |  |  |  |  |  |  |  |  |  |
| Non | 7869392 | 95.9 |  | 304616 | 89.4 |  | 790023 | 90.5 |  | 1649021 | 91.5 |
| Yes | 333296 | 4.1 |  | 36180 | 10.6 |  | 83126 | 9.5 |  | 153431 | 8.5 |
| Hospital diagnosis of obesity |  |  |  |  |  |  |  |  |  |  |  |
| Non | 8084044 | 98.6 |  | 339317 | 99.6 |  | 869376 | 99.6 |  | 1793843 | 99.5 |
| Yes | 118644 | 1.4 |  | 1479 | 0.4 |  | 3773 | 0.4 |  | 8609 | 0.5 |
| Diagnosis of cancer |  |  |  |  |  |  |  |  |  |  |  |
| Non | 7275765 | 88.7 |  | 301273 | 88.4 |  | 754559 | 86.4 |  | 1320391 | 73.3 |
| Yes | 926923 | 11.3 |  | 39523 | 11.6 |  | 118590 | 13.6 |  | 482061 | 26.7 |
| All | 8202688 | 100.0 |  | 340796 | 100.0 |  | 873149 | 100.0 |  | 1802452 | 100.0 |
| CHD: Coronary heart disease: CVD: Cardiovascular diseases; COPD: Chronic obstructive pulmonary disease. | | | | | | | | | | | |

| **Table S3.** Association between sex ratio (Males/All birth) at birth (1900-1997) and risks of fatal CVD in men, 1997-2018. | | | | | | | | | | | | |  | | | | | |
| --- | --- | --- | --- | --- | --- | --- | --- | --- | --- | --- | --- | --- | --- | --- | --- | --- | --- | --- |
|  | **Model 1** | | |  | **Model 2** | | |  | **Model 3** | | | | | |  | |  |  |
|  | HR | 95% CI | |  | HR | 95% CI | |  | HR | 95% CI | | | | |  | |  |  |
| Sex ratio at birth (Males/All birth) (ref. High) |  |  |  |  |  |  |  |  |  |  | |  | |  | |  |  |  |
| Low | 1.03 | 1.03 | 1.04 |  | 1.03 | 1.02 | 1.04 |  | 1.03 | 1.02 | | 1.04 | |  | |  |  |  |
| Middle | 1.03 | 1.02 | 1.04 |  | 1.03 | 1.02 | 1.04 |  | 1.03 | 1.02 | | 1.03 | |  | |  |  |  |
| Education level (ref. >12) |  |  |  |  |  |  |  |  |  |  | |  | |  | |  |  |  |
| <9 |  |  |  |  | 1.28 | 1.27 | 1.29 |  | 1.24 | 1.23 | | 1.25 | |  | |  |  |  |
| 10-12 |  |  |  |  | 1.20 | 1.19 | 1.21 |  | 1.18 | 1.17 | | 1.19 | |  | |  |  |  |
| Region of residence (ref. Large cities) |  |  |  |  |  |  |  |  |  |  | |  | |  | |  |  |  |
| Southern Sweden |  |  |  |  | 1.11 | 1.11 | 1.12 |  | 1.12 | 1.11 | | 1.13 | |  | |  |  |  |
| Northern Sweden |  |  |  |  | 1.21 | 1.20 | 1.22 |  | 1.20 | 1.19 | | 1.21 | |  | |  |  |  |
| Hospital diagnosis of COPD (ref. Non) |  |  |  |  |  |  |  |  | 1.18 | 1.16 | | 1.19 | |  | |  |  |  |
| Hospital diagnosis of alcoholism (ref. Non) |  |  |  |  |  |  |  |  | 2.28 | 2.25 | | 2.31 | |  | |  |  |  |
| Hospital diagnosis of diabetes (ref. Non) |  |  |  |  |  |  |  |  | 1.46 | 1.44 | | 1.47 | |  | |  |  |  |
| Hospital diagnosis of obesity (ref. Non) |  |  |  |  |  |  |  |  | 1.52 | 1.46 | | 1.60 | |  | |  |  |  |
| Diagnosis of cancer (ref. Non) |  |  |  |  |  |  |  |  | 0.59 | 0.58 | | 0.59 | |  | |  |  |  |
| CVD: Cardiovascular diseases; HR: Hazards ratio; CI: Confidence interval; COPD: Chronic obstructive pulmonary disease. | | | | | | | | | | | | |  | | | | | |
| Model 1. Crude model; Model 2: Adjusted for individual characteristics; Model 3: Fully adjusted model: Adjusted for individual characteristics and comorbidities. | | | | | | | | | | | | | | | | | |  |
|  | | | | | | | | | |  |  | |  | | | | | |
|  | | | | | | | | | |  |  | |  | | | | | |

| **Table S4**. Association between sex ratio (Males/All birth) at birth (1900-1997) and risks of fatal CHD in men, 1997-2018. | | | | | | | | | | | | | |  | | |  |  |  |
| --- | --- | --- | --- | --- | --- | --- | --- | --- | --- | --- | --- | --- | --- | --- | --- | --- | --- | --- | --- |
|  | **Model 1** | | |  | **Model 2** | | |  | **Model 3** | | | | | | | | |  | |
|  | HR | 95% CI | |  | HR | 95% CI | |  | HR | | 95% CI | | | | | | |  | |
| Sex ratio at birth (Males/All birth) (ref. High) |  |  |  |  |  |  |  |  |  | |  | | | |  | | |  | |
| Low | 1.05 | 1.04 | 1.06 |  | 1.04 | 1.03 | 1.05 |  | 1.04 | | 1.03 | | | | 1.05 | | |  | |
| Middle | 1.03 | 1.02 | 1.05 |  | 1.04 | 1.02 | 1.05 |  | 1.03 | | 1.02 | | | | 1.05 | | |  | |
| Education level (ref. >12) |  |  |  |  |  |  |  |  |  | |  | | | |  | | |  | |
| <9 |  |  |  |  | 1.41 | 1.39 | 1.43 |  | 1.36 | | 1.35 | | | | 1.38 | | |  | |
| 10-12 |  |  |  |  | 1.26 | 1.25 | 1.28 |  | 1.24 | | 1.22 | | | | 1.26 | | |  | |
| Region of residence (ref. Large cities) |  |  |  |  |  |  |  |  |  | |  | | | |  | | |  | |
| Southern Sweden |  |  |  |  | 1.12 | 1.11 | 1.13 |  | 1.13 | | 1.12 | | | | 1.14 | | |  | |
| Northern Sweden |  |  |  |  | 1.25 | 1.23 | 1.26 |  | 1.23 | | 1.22 | | | | 1.25 | | |  | |
| Hospital diagnosis of COPD (ref. Non) |  |  |  |  |  |  |  |  | 1.14 | | 1.12 | | | | 1.16 | | |  | |
| Hospital diagnosis of alcoholism (ref. Non) |  |  |  |  |  |  |  |  | 2.17 | | 2.13 | | | | 2.22 | | |  | |
| Hospital diagnosis of diabetes (ref. Non) |  |  |  |  |  |  |  |  | 1.52 | | 1.50 | | | | 1.54 | | |  | |
| Hospital diagnosis of obesity (ref. Non) |  |  |  |  |  |  |  |  | 1.36 | | 1.27 | | | | 1.46 | | |  | |
| Diagnosis of cancer (ref. Non) |  |  |  |  |  |  |  |  | 0.48 | | 0.48 | | | | 0.49 | | |  | |
| CHD: Coronary heart disease; HR: Hazards ratio; CI: Confidence interval; COPD: Chronic obstructive pulmonary disease. | | | | | | | | | | | | | | | | |  |  |  |
| Model 1. Crude model; Model 2: Adjusted for individual characteristics; Model 3: Fully adjusted model: Adjusted for individual characteristics and comorbidities. | | | | | | | | | | | | | | | | | | |  |
|  | | | | | | | | | |  | | |  | | |  | | |  |
|  | | | | | | | | | | | |  | |  | | |  |  |  |

| **Table S5**. Association between sex ratio (Males/All birth) at birth (1900-1997) and risks of non-fatal CVD in men 1997-2018. | | | | | | | | | | | | | | | | | | | | | | | | | | | | | | | | | | | |
| --- | --- | --- | --- | --- | --- | --- | --- | --- | --- | --- | --- | --- | --- | --- | --- | --- | --- | --- | --- | --- | --- | --- | --- | --- | --- | --- | --- | --- | --- | --- | --- | --- | --- | --- | --- |
|  | **Model 1** | | | | |  | | **Model 2** | | | | | | | | | |  | | | | | | **Model 3** | | | | | | | | | | |  |
|  | HR | 95% CI | | |  | | HR | | | | | 95% CI | | | | | | |  | | | | HR | | | | | 95% CI | | | | |  |  |  |
| Sex ratio at birth (Males/All birth) (ref. High) |  |  |  | |  | |  | | | |  | | |  |  | |  | | |  | | | | |  | | | |  |  |  |  |  |  |  |
| Low | 1.01 | 1.01 | | 1.02 | | | | |  | 1.01 | | | 1.01 | | | 1.02 | | | | |  | 1.01 | | | | | 1.01 | | | 1.02 | | | |  |  |
| Middle | 1.00 | 1.00 | | 1.01 | | | | |  | 1.00 | | | 1.00 | | | 1.01 | | | | |  | 1.01 | | | | | 1.00 | | | 1.01 | | | |  |  |
| Education level (ref. >12) |  |  | |  | | | | |  |  | | |  | | |  | | | | |  |  | | | | |  | | |  | | | |  |  |
| <9 |  |  | |  | | | | |  | 1.03 | | | 1.03 | | | 1.04 | | | | |  | 1.01 | | | | | 1.01 | | | 1.01 | | | |  |  |
| 10-12 |  |  | |  | | | | |  | 1.11 | | | 1.10 | | | 1.11 | | | | |  | 1.08 | | | | | 1.08 | | | 1.09 | | | |  |  |
| Region of residence (ref. Large cities) |  |  | |  | | | | |  |  | | |  | | |  | | | | |  |  | | | | |  | | |  | | | |  |  |
| Southern Sweden |  |  | |  | | | | |  | 1.03 | | | 1.03 | | | 1.03 | | | | |  | 1.04 | | | | | 1.03 | | | 1.04 | | | |  |  |
| Northern Sweden |  |  | |  | | | | |  | 1.04 | | | 1.04 | | | 1.05 | | | | |  | 1.05 | | | | | 1.04 | | | 1.05 | | | |  |  |
| Hospital diagnosis of COPD (ref. Non) |  |  | |  | | | | |  |  | | |  | | |  | | | | |  | 1.46 | | | | | 1.45 | | | 1.47 | | | |  |  |
| Hospital diagnosis of alcoholism (ref. Non) |  |  | |  | | | | |  |  | | |  | | |  | | | | |  | 1.49 | | | | | 1.48 | | | 1.50 | | | |  |  |
| Hospital diagnosis of diabetes (ref. Non) |  |  | |  | | | | |  |  | | |  | | |  | | | | |  | 1.88 | | | | | 1.86 | | | 1.89 | | | |  |  |
| Hospital diagnosis of obesity (ref. Non) |  |  | |  | | | | |  |  | | |  | | |  | | | | |  | 2.49 | | | | | 2.45 | | | 2.53 | | | |  |  |
| Diagnosis of cancer (ref. Non) |  |  | |  | | | | |  |  | | |  | | |  | | | | |  | 1.16 | | | | | 1.15 | | | 1.16 | | | |  |  |
| CVD: Cardiovascular diseases; HR: Hazards ratio; CI: Confidence interval; COPD: Chronic obstructive pulmonary disease. | | | | | | | | | | | | | | | | | | | |  | | | | | |  | | |  | |  |  |  |  |  |
| Model 1. Crude model; Model 2: Adjusted for individual characteristics; Model 3: Fully adjusted model: Adjusted for individual characteristics and comorbidities. | | | | | | | | | | | | | | | | | | | | | | | | | | | | | | | |  |  |  |  |
|  | | | | | | | | | | | | | | | | | | | |  | | | | | |  | | |  | |  |  |  |  |  |

| **Table S6.** Association between sex ratio (Males/All birth) at birth (1900-1997) and risks of non-fatal CHD in men 1997-2018. | | | | | | | | | | | | | |  | |  | |  |  |  |
| --- | --- | --- | --- | --- | --- | --- | --- | --- | --- | --- | --- | --- | --- | --- | --- | --- | --- | --- | --- | --- |
|  | **Model 1** | | |  | **Model 2** | | |  | **Model 3** | | | | | | | |  | |  |  |
|  | HR | 95% CI | |  | HR | 95% CI | |  | HR | | 95% CI | | | | | |  | |  |  |
| Sex ratio at birth (Males/All birth) (ref. High) |  |  |  |  |  |  |  |  |  | |  | |  | | | |  | |  | |
| Low | 1.04 | 1.03 | 1.05 |  | 1.03 | 1.02 | 1.05 |  | 1.03 | | 1.02 | | 1.04 | | | |  | |  | |
| Middle | 1.02 | 1.01 | 1.03 |  | 1.02 | 1.01 | 1.03 |  | 1.02 | | 1.01 | | 1.03 | | | |  | |  | |
| Education level (ref. >12) |  |  |  |  |  |  |  |  |  | |  | |  | | | |  | |  | |
| <9 |  |  |  |  | 1.22 | 1.21 | 1.23 |  | 1.20 | | 1.19 | | 1.21 | | | |  | |  | |
| 10-12 |  |  |  |  | 1.21 | 1.20 | 1.22 |  | 1.19 | | 1.18 | | 1.20 | | | |  | |  | |
| Region of residence (ref. Large cities) |  |  |  |  |  |  |  |  |  | |  | |  | | | |  | |  | |
| Southern Sweden |  |  |  |  | 1.12 | 1.12 | 1.13 |  | 1.13 | | 1.12 | | 1.14 | | | |  | |  | |
| Northern Sweden |  |  |  |  | 1.27 | 1.26 | 1.29 |  | 1.27 | | 1.26 | | 1.28 | | | |  | |  | |
| Hospital diagnosis of COPD (ref. Non) |  |  |  |  |  |  |  |  | 1.50 | | 1.48 | | 1.52 | | | |  | |  | |
| Hospital diagnosis of alcoholism (ref. Non) |  |  |  |  |  |  |  |  | 1.08 | | 1.06 | | 1.10 | | | |  | |  | |
| Hospital diagnosis of diabetes (ref. Non) |  |  |  |  |  |  |  |  | 2.00 | | 1.97 | | 2.02 | | | |  | |  | |
| Hospital diagnosis of obesity (ref. Non) |  |  |  |  |  |  |  |  | 1.79 | | 1.71 | | 1.87 | | | |  | |  | |
| Diagnosis of cancer (ref. Non) |  |  |  |  |  |  |  |  | 1.03 | | 1.02 | | 1.04 | | | |  | |  | |
| CHD: Coronary heart disease; HR: Hazards ratio; CI: Confidence interval; COPD: Chronic obstructive pulmonary disease. | | | | | | | | | | | | | | | | | | |  |  |
| Model 1. Crude model; Model 2: Adjusted for individual characteristics; Model 3: Fully adjusted model: Adjusted for individual characteristics and comorbidities. | | | | | | | | | | | | | | | | | | | |  |
|  | | | | | | | | | |  | |  | | |  | | | | |  |
|  | | | | | | | | | | |  | |  | | | |  | |  | |

| **Table S7**. Association between sex ratio (Males/All birth) at birth (1900-1997) and risks of fatal CVD in women 1997-2018. | | | | | | | | | | | | | | |  | |  |  |  |
| --- | --- | --- | --- | --- | --- | --- | --- | --- | --- | --- | --- | --- | --- | --- | --- | --- | --- | --- | --- |
|  | **Model 1** | | |  | **Model 2** | | |  | **Model 3** | | | | | | |  | | |  |
|  | HR | 95% CI | |  | HR | 95% CI | |  | HR | | 95% CI | | | | |  | | |  |
| Sex ratio at birth (Males/All birth) (ref. High) |  |  |  |  |  |  |  |  |  | |  | |  | | |  | | |  |
| Low | 1.02 | 1.01 | 1.02 |  | 1.01 | 1.00 | 1.02 |  | 1.01 | | 1.00 | | 1.02 | | |  | | |  |
| Middle | 1.02 | 1.01 | 1.03 |  | 1.02 | 1.01 | 1.03 |  | 1.02 | | 1.01 | | 1.03 | | |  | | |  |
| Education level (ref. >12) |  |  |  |  |  |  |  |  |  | |  | |  | | |  | | |  |
| <9 |  |  |  |  | 1.14 | 1.13 | 1.15 |  | 1.13 | | 1.12 | | 1.14 | | |  | | |  |
| 10-12 |  |  |  |  | 1.00 | 0.99 | 1.01 |  | 1.00 | | 0.99 | | 1.01 | | |  | | |  |
| Region of residence (ref. Large cities) |  |  |  |  |  |  |  |  |  | |  | |  | | |  | | |  |
| Southern Sweden |  |  |  |  | 1.14 | 1.13 | 1.14 |  | 1.14 | | 1.13 | | 1.14 | | |  | | |  |
| Northern Sweden |  |  |  |  | 1.20 | 1.19 | 1.21 |  | 1.19 | | 1.18 | | 1.20 | | |  | | |  |
| Hospital diagnosis of COPD (ref. Non) |  |  |  |  |  |  |  |  | 1.28 | | 1.27 | | 1.30 | | |  | | |  |
| Hospital diagnosis of alcoholism (ref. Non) |  |  |  |  |  |  |  |  | 2.41 | | 2.35 | | 2.48 | | |  | | |  |
| Hospital diagnosis of diabetes (ref. Non) |  |  |  |  |  |  |  |  | 1.57 | | 1.55 | | 1.59 | | |  | | |  |
| Hospital diagnosis of obesity (ref. Non) |  |  |  |  |  |  |  |  | 1.46 | | 1.40 | | 1.53 | | |  | | |  |
| Diagnosis of cancer (ref. Non) |  |  |  |  |  |  |  |  | 0.67 | | 0.66 | | 0.68 | | |  | | |  |
| CVD: Cardiovascular diseases; HR: Hazards ratio; CI: Confidence interval; COPD: Chronic obstructive pulmonary disease. | | | | | | | | | | | | | | |  | | | | |
| Model 1. Crude model; Model 2: Adjusted for individual characteristics; Model 3: Fully adjusted model: Adjusted for individual characteristics and comorbidities. | | | | | | | | | | | | | | | | | |  |  |
|  | | | | | | | | | |  | |  | |  | | | |  |  |
|  | | | | | | | | | | | | | | | | | | | |

| **Table S8.** Association between sex ratio (Males/All birth) at birth (1900-1997) and risks of fatal CHD in women 1997-2018. | | | | | | | | | | | | | |  | | |  | |  |  |
| --- | --- | --- | --- | --- | --- | --- | --- | --- | --- | --- | --- | --- | --- | --- | --- | --- | --- | --- | --- | --- |
|  | **Model 1** | | |  | **Model 2** | | |  | | **Model 3** | | | | | | | |  | | |
|  | HR | 95% CI | |  | HR | 95% CI | |  | | HR | | | 95% CI | | | | |  | | |
| Sex ratio at birth (Males/All birth) (ref. High) |  |  |  |  |  |  |  |  | |  | | |  | |  | | |  | | |
| Low | 1.04 | 1.02 | 1.05 |  | 1.03 | 1.02 | 1.05 |  | | 1.03 | | | 1.02 | | 1.05 | | |  | | |
| Middle | 1.04 | 1.03 | 1.05 |  | 1.04 | 1.03 | 1.06 |  | | 1.04 | | | 1.03 | | 1.06 | | |  | | |
| Education level (ref. >12) |  |  |  |  |  |  |  |  | |  | | |  | |  | | |  | | |
| <9 |  |  |  |  | 1.20 | 1.18 | 1.22 |  | | 1.19 | | | 1.17 | | 1.20 | | |  | | |
| 10-12 |  |  |  |  | 0.98 | 0.96 | 1.00 |  | | 0.98 | | | 0.96 | | 1.00 | | |  | | |
| Region of residence (ref. Large cities) |  |  |  |  |  |  |  |  | |  | | |  | |  | | |  | | |
| Southern Sweden |  |  |  |  | 1.15 | 1.13 | 1.16 |  | | 1.14 | | | 1.13 | | 1.16 | | |  | | |
| Northern Sweden |  |  |  |  | 1.26 | 1.24 | 1.27 |  | | 1.23 | | | 1.22 | | 1.25 | | |  | | |
| Hospital diagnosis of COPD (ref. Non) |  |  |  |  |  |  |  |  | | 1.33 | | | 1.30 | | 1.35 | | |  | | |
| Hospital diagnosis of alcoholism (ref. Non) |  |  |  |  |  |  |  |  | | 2.29 | | | 2.18 | | 2.40 | | |  | | |
| Hospital diagnosis of diabetes (ref. Non) |  |  |  |  |  |  |  |  | | 1.82 | | | 1.79 | | 1.85 | | |  | | |
| Hospital diagnosis of obesity (ref. Non) |  |  |  |  |  |  |  |  | | 1.41 | | | 1.31 | | 1.53 | | |  | | |
| Diagnosis of cancer (ref. Non) |  |  |  |  |  |  |  |  | | 0.53 | | | 0.52 | | 0.54 | | |  | | |
| CHD: Coronary heart disease; HR: Hazards ratio; CI: Confidence interval; COPD: Chronic obstructive pulmonary disease. | | | | | | | | | | | | | | | | |  | |  |  |
| Model 1. Crude model; Model 2: Adjusted for individual characteristics; Model 3: Fully adjusted model: Adjusted for individual characteristics and comorbidities. | | | | | | | | | | | | | | | | | | | |  |
|  | | | | | | | | |  | |  | | | | |  | | | |  |
|  | | | | | | | | | | | |  | |  | | |  | |  |  |

| **Table S9**. Association between sex ratio (Males/All birth) at birth (1900-1997) and risks of non-fatal CVD in women, 1997-2018. | | | | | | | | | | | | | | |  |  |
| --- | --- | --- | --- | --- | --- | --- | --- | --- | --- | --- | --- | --- | --- | --- | --- | --- |
|  | **Model 1** | | |  | **Model 2** | | |  | **Model 3** | | | | | |  |  |
|  | HR | 95% CI | |  | HR | 95% CI | |  | HR | 95% CI | | | | |  |  |
| Sex ratio at birth (Males/All birth) (ref. High) |  |  |  |  |  |  |  |  |  |  | | |  | |  |  |
| Low | 1.00 | 1.00 | 1.01 |  | 1.00 | 1.00 | 1.01 |  | 1.00 | 1.00 | | | 1.00 | |  |  |
| Middle | 0.99 | 0.99 | 1.00 |  | 1.00 | 0.99 | 1.00 |  | 1.00 | 0.99 | | | 1.00 | |  |  |
| Education level (ref. >12) |  |  |  |  |  |  |  |  |  |  | | |  | |  |  |
| <9 |  |  |  |  | 1.06 | 1.06 | 1.07 |  | 1.04 | 1.03 | | | 1.04 | |  |  |
| 10-12 |  |  |  |  | 1.13 | 1.13 | 1.14 |  | 1.10 | 1.10 | | | 1.11 | |  |  |
| Region of residence (ref. Large cities) |  |  |  |  |  |  |  |  |  |  | | |  | |  |  |
| Southern Sweden |  |  |  |  | 1.02 | 1.01 | 1.02 |  | 1.02 | 1.02 | | | 1.02 | |  |  |
| Northern Sweden |  |  |  |  | 1.01 | 1.01 | 1.02 |  | 1.02 | 1.01 | | | 1.02 | |  |  |
| Hospital diagnosis of COPD (ref. Non) |  |  |  |  |  |  |  |  | 1.59 | 1.58 | | | 1.60 | |  |  |
| Hospital diagnosis of alcoholism (ref. Non) |  |  |  |  |  |  |  |  | 1.51 | 1.49 | | | 1.53 | |  |  |
| Hospital diagnosis of diabetes (ref. Non) |  |  |  |  |  |  |  |  | 1.82 | 1.81 | | | 1.84 | |  |  |
| Hospital diagnosis of obesity (ref. Non) |  |  |  |  |  |  |  |  | 2.05 | 2.03 | | | 2.08 | |  |  |
| Diagnosis of cancer (ref. Non) |  |  |  |  |  |  |  |  | 1.26 | 1.26 | | | 1.27 | |  |  |
| CVD: Cardiovascular diseases; HR: Hazards ratio; CI: Confidence interval; COPD: Chronic obstructive pulmonary disease. | | | | | | | | | | | | | | | |  |
| Model 1. Crude model; Model 2: Adjusted for individual characteristics; Model 3: Fully adjusted model: Adjusted for individual characteristics and comorbidities. | | | | | | | | | | | | | | | | |
|  | | | | | | | | | | |  |  | |  | | |
|  | | | | | | | | | | | | | | | |  |

| **Table S10.** Association between sex ratio (Males/All birth) at birth (1900-1997) and risks of non-fatal CHD in women, 1997-2018. | | | | | | | | | | | | | | | |  |  |
| --- | --- | --- | --- | --- | --- | --- | --- | --- | --- | --- | --- | --- | --- | --- | --- | --- | --- |
|  | **Model 1** | | |  | **Model 2** | | |  | **Model 3** | | | | | | |  |  |
|  | HR | 95% CI | |  | HR | 95% CI | |  | HR | 95% CI | | | | | |  |  |
| Sex ratio at birth (Males/All birth) (ref. High) |  |  |  |  |  |  |  |  |  |  | | |  |  |  |  |  |
| Low | 1.03 | 1.02 | 1.05 |  | 1.03 | 1.01 | 1.04 |  | 1.02 | 1.01 | | | 1.04 |  |  |  |  |
| Middle | 1.02 | 1.01 | 1.04 |  | 1.03 | 1.01 | 1.04 |  | 1.03 | 1.01 | | | 1.04 |  |  |  |  |
| Education level (ref. >12) |  |  |  |  |  |  |  |  |  |  | | |  |  |  |  |  |
| <9 |  |  |  |  | 1.41 | 1.39 | 1.43 |  | 1.37 | 1.35 | | | 1.38 |  |  |  |  |
| 10-12 |  |  |  |  | 1.28 | 1.27 | 1.30 |  | 1.25 | 1.23 | | | 1.27 |  |  |  |  |
| Region of residence (ref. Large cities) |  |  |  |  |  |  |  |  |  |  | | |  |  |  |  |  |
| Southern Sweden |  |  |  |  | 1.12 | 1.10 | 1.13 |  | 1.12 | 1.10 | | | 1.13 |  |  |  |  |
| Northern Sweden |  |  |  |  | 1.28 | 1.26 | 1.30 |  | 1.27 | 1.25 | | | 1.29 |  |  |  |  |
| Hospital diagnosis of COPD (ref. Non) |  |  |  |  |  |  |  |  | 1.88 | 1.84 | | | 1.91 |  |  |  |  |
| Hospital diagnosis of alcoholism (ref. Non) |  |  |  |  |  |  |  |  | 1.18 | 1.13 | | | 1.24 |  |  |  |  |
| Hospital diagnosis of diabetes (ref. Non) |  |  |  |  |  |  |  |  | 2.34 | 2.31 | | | 2.38 |  |  |  |  |
| Hospital diagnosis of obesity (ref. Non) |  |  |  |  |  |  |  |  | 1.70 | 1.63 | | | 1.78 |  |  |  |  |
| Diagnosis of cancer (ref. Non) |  |  |  |  |  |  |  |  | 0.99 | 0.98 | | | 1.00 |  |  |  |  |
| CHD: Coronary heart disease; HR: Hazards ratio; CI: Confidence interval; COPD: Chronic obstructive pulmonary disease. | | | | | | | | | | | | | | | | |  |
| Model 1. Crude model; Model 2: Adjusted for individual characteristics; Model 3: Fully adjusted model: Adjusted for individual characteristics and comorbidities. | | | | | | | | | | | | | | | | | |
|  | | | | | | | | | | |  |  | | |  | | |
|  | | | | | | | | | | | | | | | | |  |

| **Table S11**. Association between sex ratio (Males/All birth) at birth (1900-1997) and total mortality in men 1997-2018. | | | | | | | | | | | |  | | |  | |  |  |
| --- | --- | --- | --- | --- | --- | --- | --- | --- | --- | --- | --- | --- | --- | --- | --- | --- | --- | --- |
|  | **Model 1** | | |  | **Model 2** | | |  | **Model 3** | | | | | | |  | |  |
|  | HR | 95% CI | |  | HR | 95% CI | |  | HR | 95% CI | | | | | |  | |  |
| Sex ratio at birth (Males/All birth) (ref. High) |  |  |  |  |  |  |  |  |  |  | | | |  | |  | |  |
| Low | 1.03 | 1.02 | 1.03 |  | 1.02 | 1.01 | 1.03 |  | 1.02 | 1.01 | | | | 1.03 | |  | |  |
| Middle | 1.02 | 1.01 | 1.02 |  | 1.02 | 1.01 | 1.02 |  | 1.02 | 1.01 | | | | 1.02 | |  | |  |
| Education level (ref. >12) |  |  |  |  |  |  |  |  |  |  | | | |  | |  | |  |
| <9 |  |  |  |  | 1.23 | 1.22 | 1.24 |  | 1.22 | 1.22 | | | | 1.23 | |  | |  |
| 10-12 |  |  |  |  | 1.19 | 1.18 | 1.20 |  | 1.16 | 1.16 | | | | 1.17 | |  | |  |
| Region of residence (ref. Large cities) |  |  |  |  |  |  |  |  |  |  | | | |  | |  | |  |
| Southern Sweden |  |  |  |  | 1.07 | 1.06 | 1.07 |  | 1.08 | 1.08 | | | | 1.09 | |  | |  |
| Northern Sweden |  |  |  |  | 1.14 | 1.13 | 1.15 |  | 1.17 | 1.16 | | | | 1.18 | |  | |  |
| Hospital diagnosis of COPD (ref. Non) |  |  |  |  |  |  |  |  | 1.26 | 1.25 | | | | 1.27 | |  | |  |
| Hospital diagnosis of alcoholism (ref. Non) |  |  |  |  |  |  |  |  | 2.83 | 2.80 | | | | 2.86 | |  | |  |
| Hospital diagnosis of diabetes (ref. Non) |  |  |  |  |  |  |  |  | 1.29 | 1.28 | | | | 1.30 | |  | |  |
| Hospital diagnosis of obesity (ref. Non) |  |  |  |  |  |  |  |  | 1.22 | 1.18 | | | | 1.26 | |  | |  |
| Diagnosis of cancer (ref. Non) |  |  |  |  |  |  |  |  | 1.36 | 1.35 | | | | 1.37 | |  | |  |
| HR: Hazards ratio; CI: Confidence interval; COPD: Chronic obstructive pulmonary disease. | | | | | | | | | | | | | | |  | |  |  |
| Model 1. Crude model; Model 2: Adjusted for individual characteristics; Model 3: Fully adjusted model: Adjusted for individual characteristics and comorbidities. | | | | | | | | | | | | | | | | | | |
|  | | | | | | | | | | |  | |  | | |  | | |
|  | | | | | | | | | | | | | | | | |  |  |

| **Table S12**. Association between sex ratio (Males/All birth) at birth (1900-1997) and risks of total mortality in women 1997-2018. | | | | | | | | | | | | |  | | |  | | | | | |
| --- | --- | --- | --- | --- | --- | --- | --- | --- | --- | --- | --- | --- | --- | --- | --- | --- | --- | --- | --- | --- | --- |
|  | **Model 1** | | |  | **Model 2** | | |  | **Model 3** | | | | | | | | |  | | |  |
|  | HR | 95% CI | |  | HR | 95% CI | |  | HR | | 95% CI | | | | | | |  | | |  |
| Sex ratio at birth (Males/All birth) (ref. High) |  |  |  |  |  |  |  |  |  | |  | | |  | | |  | | |  |  |
| Low | 1.01 | 1.00 | 1.02 |  | 1.01 | 1.00 | 1.01 |  | 1.00 | | 1.00 | | | 1.01 | | |  | | |  |  |
| Middle | 1.01 | 1.00 | 1.01 |  | 1.01 | 1.00 | 1.01 |  | 1.01 | | 1.01 | | | 1.02 | | |  | | |  |  |
| Birth (year) |  |  |  |  |  |  |  |  |  | |  | | |  | | |  | | |  |  |
| Education level (ref. >12) |  |  |  |  | 1.14 | 1.14 | 1.15 |  | 1.13 | | 1.12 | | | 1.13 | | |  | | |  |  |
| <9 |  |  |  |  | 1.05 | 1.05 | 1.06 |  | 1.03 | | 1.02 | | | 1.03 | | |  | | |  |  |
| 10-12 |  |  |  |  |  |  |  |  |  | |  | | |  | | |  | | |  |  |
| Region of residence (ref. Large cities) |  |  |  |  | 1.07 | 1.07 | 1.08 |  | 1.08 | | 1.08 | | | 1.09 | | |  | | |  |  |
| Southern Sweden |  |  |  |  | 1.14 | 1.14 | 1.15 |  | 1.17 | | 1.16 | | | 1.17 | | |  | | |  |  |
| Northern Sweden |  |  |  |  |  |  |  |  | 1.42 | | 1.41 | | | 1.43 | | |  | | |  |  |
| Hospital diagnosis of COPD (ref. Non) |  |  |  |  |  |  |  |  | 2.93 | | 2.88 | | | 2.97 | | |  | | |  |  |
| Hospital diagnosis of alcoholism (ref. Non) |  |  |  |  |  |  |  |  | 1.37 | | 1.36 | | | 1.39 | | |  | | |  |  |
| Hospital diagnosis of diabetes (ref. Non) |  |  |  |  |  |  |  |  | 1.17 | | 1.13 | | | 1.20 | | |  | | |  |  |
| Hospital diagnosis of obesity (ref. Non) |  |  |  |  |  |  |  |  | 1.65 | | 1.64 | | | 1.66 | | |  | | |  |  |
| Diagnosis of cancer (ref. Non) | 1.01 | 1.00 | 1.02 |  | 1.01 | 1.00 | 1.01 |  | 1.00 | | 1.00 | | | 1.01 | | |  | | |  |  |
| HR: Hazards ratio; CI: Confidence interval; COPD: Chronic obstructive pulmonary disease. | | | | | | | | | | | | | | | |  | | | | | |
| Model 1. Crude model; Model 2: Adjusted for individual characteristics; Model 3: Fully adjusted model: Adjusted for individual characteristics and comorbidities. | | | | | | | | | | | | | | | | | | |  |  |  |
|  | | | | | | | | | |  | |  | | |  | | | |  |  |  |
|  | | | | | | | | | | | | | | | | | | | | | |

| **Table S13**. Association between sex ratio (M/All birth) at birth (1932-1997) and risks of *non-fatal* CVD and CHD, 1997-2018, adjusted for family size and maternal age at birth (N=6 563 604). | | | | | | | | |
| --- | --- | --- | --- | --- | --- | --- | --- | --- |
|  | **Men** | | |  | **Women** | | | |
|  | HR* | 95% CI | |  | HR* | 95% CI | | |
| **CVD** |  |  |  |  |  |  |  | |
| Sex ratio at birth (Males/All birth) (ref. High) |  |  |  |  |  |  |  | |
| Low | 1.01 | 1.00 | 1.01 |  | 1.01 | 1.00 | 1.02 | |
| Middle | 1.01 | 1.00 | 1.01 |  | 1.00 | 0.99 | 1.00 | |
| **CHD** |  |  |  |  |  |  |  | |
| Sex ratio at birth (Males/All birth) (ref. High) |  |  |  |  |  |  |  | |
| Low | 0.95 | 0.94 | 0.97 |  | 0.93 | 0.91 | 0.95 | |
| Middle | 0.99 | 0.98 | 1.01 |  | 0.98 | 0.96 | 0.99 | |
| CVD: Cardiovascular diseases; CHD: Coronary heart disease; HR: Hazards ratio; CI: Confidence interval. | | | | | | | | |
| * Fully adjusted: Adjusted for individual characteristics and comorbidities. | | | | | | | |  |

| **Table S14**. Association between sex ratio (males/all births) at birth (1932-1997) and risks of fatal CVD, fatal CHD, and total mortality 1997-2018, adjusted for family size and maternal age at birth (N=6 563 604). | | | | | | | |
| --- | --- | --- | --- | --- | --- | --- | --- |
|  | **Men** | | |  | **Women** | | |
|  | HR* | 95% CI | |  | HR* | 95% CI | |
| **Fatal CVD** |  |  |  |  |  |  |  |
| Sex ratio at birth (Males/All birth) (ref. High) |  |  |  |  |  |  |  |
| Low | 0.93 | 0.92 | 0.95 |  | 0.96 | 0.94 | 0.99 |
| Middle | 0.99 | 0.97 | 1.00 |  | 0.99 | 0.97 | 1.01 |
| **Fatal CHD** |  |  |  |  |  |  |  |
| Sex ratio at birth (Males/All birth) (ref. High) |  |  |  |  |  |  |  |
| Low | 0.92 | 0.90 | 0.94 |  | 0.91 | 0.87 | 0.95 |
| Middle | 0.99 | 0.97 | 1.01 |  | 0.98 | 0.95 | 1.02 |
| **Total mortality** |  |  |  |  |  |  |  |
| Sex ratio at birth (Males/All birth) (ref. High) |  |  |  |  |  |  |  |
| Low | 0.98 | 0.97 | 0.99 |  | 1.01 | 0.99 | 1.02 |
| Middle | 1.01 | 1.00 | 1.02 |  | 1.01 | 1.00 | 1.02 |
| CVD: Cardiovascular diseases; CHD: Coronary heart disease; HR: Hazards ratio; CI: Confidence interval. | | | | | | | |
| * Fully adjusted: Adjusted for individual characteristics and comorbidities. | | | | | | | |

**Table S15**. Numbers of births and outcomes during two separate time periods of birth and follow-up.

| **Period 1** | |  | **Period 2** | |
| --- | --- | --- | --- | --- |
| Birth year 1900-1934 | |  | Birth year 1935-1980 | |
| Follow-up 1964-1986 | |  | Follow-up 1997-2018 | |
| Study population | 3 275 125 |  | Study population | 4 769 492 |
| Number of events | |  | Number of events: | |
| CVD | 773 213 |  | CVD | 1 503 208 |
| CHD | 260 151 |  | CHD | 209 279 |
|  |  |  |  |  |
| Fatal CVD | 526 584 |  | Fatal CVD | 126 256 |
| Fatal CHD | 333 678 |  | Fatal CHD | 63 128 |
| Total mortality | 2 981 634 |  | Total mortality | 439 983 |

| **Table S16**. Association between tertiles of sex ratio (males/all birth) at birth and risks of cardiovascular disorders and mortality during two time periods (1900-1934 vs. 1935-1980) | | | | | | | |
| --- | --- | --- | --- | --- | --- | --- | --- |
|  | **Period 1** | | |  | **Period 2** | | |
|  | HR* | 95% CI | |  | HR* | 95% CI | |
| **Men non-fatal CVD** |  |  |  |  |  |  |  |
| Sex ratio at birth (Males/All birth) (ref. High) |  |  |  |  |  |  |  |
| Low | 0.92 | 0.92 | 0.93 |  | 0.99 | 0.98 | 0.99 |
| Middle | 0.98 | 0.98 | 0.99 |  | 0.99 | 0.99 | 1.00 |
| **Men non-fatal CHD** |  |  |  |  |  |  |  |
| Sex ratio at birth (Males/All birth) (ref. High) |  |  |  |  |  |  |  |
| Low | 0.96 | 0.94 | 0.97 |  | 1.09 | 1.08 | 1.11 |
| Middle | 1.00 | 0.99 | 1.01 |  | 1.03 | 1.02 | 1.04 |
|  |  |  |  |  |  |  |  |
| **Women non-fatal CVD** |  |  |  |  |  |  |  |
| Sex ratio at birth (Males/All birth) (ref. High) |  |  |  |  |  |  |  |
| Low | 0.88 | 0.87 | 0.89 |  | 0.99 | 0.98 | 1.00 |
| Middle | 0.97 | 0.96 | 0.98 |  | 1.00 | 0.99 | 1.01 |
| **Women non-fatal CHD** |  |  |  |  |  |  |  |
| Sex ratio at birth (Males/All birth) (ref. High) |  |  |  |  |  |  |  |
| Low | 0.88 | 0.87 | 0.90 |  | 1.07 | 1.05 | 1.10 |
| Middle | 0.96 | 0.95 | 0.98 |  | 1.01 | 0.99 | 1.04 |
|  |  |  |  |  |  |  |  |
| **Men fatal CVD** |  |  |  |  |  |  |  |
| Sex ratio at birth (Males/All birth) (ref. High) |  |  |  |  |  |  |  |
| Low | 1.07 | 1.06 | 1.08 |  | 1.01 | 1.00 | 1.03 |
| Middle | 1.04 | 1.03 | 1.05 |  | 0.97 | 0.96 | 0.99 |
| **Men fatal CHD** |  |  |  |  |  |  |  |
| Sex ratio at birth (Males/All birth) (ref. High) |  |  |  |  |  |  |  |
| Low | 1.09 | 1.08 | 1.10 |  | 1.03 | 1.01 | 1.06 |
| Middle | 1.04 | 1.03 | 1.05 |  | 0.98 | 0.96 | 1.01 |
| **Men total mortality** |  |  |  |  |  |  |  |
| Sex ratio at birth (Males/All birth) (ref. High) |  |  |  |  |  |  |  |
| Low | 0.92 | 0.92 | 0.92 |  | 0.98 | 0.97 | 1.00 |
| Middle | 0.99 | 0.99 | 1.00 |  | 0.98 | 0.97 | 0.99 |
|  |  |  |  |  |  |  |  |
| **Women fatal CVD** |  |  |  |  |  |  |  |
| Sex ratio at birth (Males/All birth) (ref. High) |  |  |  |  |  |  |  |
| Low | 1.09 | 1.08 | 1.11 |  | 0.99 | 0.96 | 1.01 |
| Middle | 1.04 | 1.03 | 1.05 |  | 0.98 | 0.95 | 1.00 |
| **Women fatal CHD** |  |  |  |  |  |  |  |
| Sex ratio at birth (Males/All birth) (ref. High) |  |  |  |  |  |  |  |
| Low | 1.15 | 1.12 | 1.17 |  | 0.96 | 0.92 | 1.00 |
| Middle | 1.05 | 1.03 | 1.07 |  | 0.95 | 0.91 | 0.99 |
| **Women total mortality** |  |  |  |  |  |  |  |
| Sex ratio at birth (Males/All birth) (ref. High) |  |  |  |  |  |  |  |
| Low | 0.79 | 0.78 | 0.79 |  | 0.97 | 0.96 | 0.99 |
| Middle | 0.95 | 0.95 | 0.96 |  | 0.99 | 0.98 | 1.01 |
| *: Fully adjusted: Adjusted for individual characteristics and comorbidities. | | | | | | | |

| **Table S17**. Associations between tertiles of sex ratio (males/all birth) at birth (1900-1997) and risks (Hazard ratios, HR) of disease by gender, and risk ratio (RR) for men compared to women. | | | | | | | | | | | | | | | | | | | | | | | |  |  |  |  |  |  |  |  |  |  |
| --- | --- | --- | --- | --- | --- | --- | --- | --- | --- | --- | --- | --- | --- | --- | --- | --- | --- | --- | --- | --- | --- | --- | --- | --- | --- | --- | --- | --- | --- | --- | --- | --- | --- |
|  | Men | | | |  | | Women | | | | | | |  | | Risk ratio (RR) of men compared to women | | | | | | | |  | | |  | | |  | |  | |
|  | HR* | 95% CI | | |  | | HR* | | 95% CI | | |  | | | RR | | | 95% CI | | | |  | | |  | | |  | | |  | |  |
| **Non-fatal CVD** |  |  |  |  | |  | |  | |  |  | |  | | | |  | |  |  |  | |  | | |  | | |  |  |  |  |  |
| Low to high | 1.01 | 1.01 | 1.02 |  | | 1.00 | | 1.00 | | 1.00 |  | | 1.01 | | | | 1.01 | | 1.01 |  |  | |  | | |  | | |  |  |  |  |  |
| Middle to high | 1.01 | 1.00 | 1.01 |  | | 1.00 | | 0.99 | | 1.00 |  | | 1.01 | | | | 1.01 | | 1.01 |  |  | |  | | |  | | |  |  |  |  |  |
| **Non-fatal CHD** |  |  |  |  | |  | |  | |  |  | |  | | | |  | |  |  |  | |  | | |  | | |  |  |  |  |  |
| Low to high | 1.03 | 1.02 | 1.04 |  | | 1.02 | | 1.01 | | 1.04 |  | | 1.01 | | | | 1.00 | | 1.02 |  |  | |  | | |  | | |  |  |  |  |  |
| Middle to high | 1.02 | 1.01 | 1.03 |  | | 1.03 | | 1.01 | | 1.04 |  | | 0.99 | | | | 0.98 | | 1.00 |  |  | |  | | |  | | |  |  |  |  |  |
| **Fatal CVD** |  |  |  |  | |  | |  | |  |  | |  | | | |  | |  |  |  | |  | | |  | | |  |  |  |  |  |
| Low to high | 1.03 | 1.02 | 1.04 |  | | 1.01 | | 1.00 | | 1.02 |  | | 1.02 | | | | 1.01 | | 1.03 |  |  | |  | | |  | | |  |  |  |  |  |
| Middle to high | 1.03 | 1.02 | 1.03 |  | | 1.02 | | 1.01 | | 1.03 |  | | 1.01 | | | | 1.00 | | 1.02 |  |  | |  | | |  | | |  |  |  |  |  |
| **Fatal CHD** |  |  |  |  | |  | |  | |  |  | |  | | | |  | |  |  |  | |  | | |  | | |  |  |  |  |  |
| Low to high | 1.04 | 1.03 | 1.05 |  | | 1.03 | | 1.02 | | 1.05 |  | | 1.01 | | | | 1.00 | | 1.02 |  |  | |  | | |  | | |  |  |  |  |  |
| Middle to high | 1.03 | 1.02 | 1.05 |  | | 1.04 | | 1.03 | | 1.06 |  | | 0.99 | | | | 0.98 | | 1.00 |  |  | |  | | |  | | |  |  |  |  |  |
| **Total mortality** |  |  |  |  | |  | |  | |  |  | |  | | | |  | |  |  |  | |  | | |  | | |  |  |  |  |  |
| Low to high | 1.02 | 1.01 | 1.03 |  | | 1.00 | | 1.00 | | 1.01 |  | | 1.02 | | | | 1.01 | | 1.03 |  |  | |  | | |  | | |  |  |  |  |  |
| Middle to high | 1.02 | 1.01 | 1.02 |  | | 1.01 | | 1.01 | | 1.02 |  | | 1.01 | | | | 1.01 | | 1.01 |  |  | |  | | |  | | |  |  |  |  |  |
| *: Fully adjusted: Adjusted for individual characteristics and comorbidities. | | | | | | | | | | | | | | | | | | | |  |  | |  | | |  | | |  |  |  |  |  |
